# The Diagnostic Performance of Diffusion Kurtosis Imaging in the Characterization of Breast Tumors: A Meta-Analysis

**DOI:** 10.3389/fonc.2020.575272

**Published:** 2020-10-27

**Authors:** Zhipeng Li, Xinming Li, Chuan Peng, Wei Dai, Haitao Huang, Xie Li, Chuanmiao Xie, Jianye Liang

**Affiliations:** ^1^Department of Medical Imaging, Sun Yat-sen University Cancer Center, State Key Laboratory of Oncology in South China, Collaborative Innovation Center for Cancer Medicine, Guangzhou, China; ^2^Department of Radiology, Zhujiang Hospital, Southern Medical University, Guangzhou, China; ^3^Department of Radiology, Maoming People's Hospital, Maoming, China; ^4^Medical Imaging Center, The First Affiliated Hospital of Jinan University, Guangzhou, China

**Keywords:** diffusion kurtosis imaging, non-Gaussian, breast tumor, magnetic resonance imaging, meta-analysis

## Abstract

**Rationale and Objectives:** Diffusion kurtosis imaging (DKI) is a promising imaging technique, but the results regarding the diagnostic performance of DKI in the characterization and classification of breast tumors are inconsistent among published studies. This study aimed to pool all published results to provide more robust evidence of the differential diagnosis between malignant and benign breast tumors using DKI.

**Methods:** Studies on the differential diagnosis of breast tumors using DKI-derived parameters were systemically retrieved from PubMed, Embase, and Web of Science without a time limit. Review Manager 5.3 was used to calculate the standardized mean differences (SMDs) and 95% confidence intervals of the mean kurtosis (MK), mean diffusivity (MD), and apparent diffusion coefficient (ADC). Stata 12.0 was used to pool the sensitivity, specificity, and diagnostic odds ratio (DOR) as well as the publication bias and heterogeneity of each parameter. Fagan's nomograms were plotted to predict the post-test probabilities.

**Results:** Thirteen studies including 867 malignant and 460 benign breast lesions were analyzed. Most of the included studies showed a low to unclear risk of bias and low concerns regarding applicability. Breast cancer showed a higher MK (SMD = 1.23, *P* < 0.001) but a lower MD (SMD = −1.29, *P* < 0.001) and ADC (SMD = −1.21, *P* < 0.001) than benign tumors. The MK (SMD = −1.36, *P* = 0.006) rather than the MD (SMD = 0.29, *P* = 0.20) or ADC (SMD = 0.26, *P* = 0.24) can further differentiate invasive ductal carcinoma from ductal carcinoma *in situ*. The DKI-derived MK (sensitivity = 90%, specificity = 88%, DOR = 66) and MD (sensitivity = 86% and specificity = 88%, DOR = 46) demonstrated superior diagnostic performance and post-test probability (65, 64, and 56% for MK, MD, and ADC) in differentiating malignant from benign breast lesions, with a higher sensitivity and specificity than the DWI-derived ADC (sensitivity = 85% and specificity = 83%, DOR = 29).

**Conclusion:** The DKI-derived MK and MD demonstrate a comparable diagnostic performance in the discrimination of breast tumors based on their microstructures and non-Gaussian characteristics. The MK can further differentiate invasive ductal carcinoma from ductal carcinoma *in situ*.

## Introduction

Breast cancer has become the most common cancer in females and accounted for 30% of estimated new cases in 2020. However, the 5-year relative survival rate is high in breast cancer (90%), which is mainly attributed to early detection through screening. Recently, a recommendation produced by EUSOMA and endorsed by ECCO emphasized the importance of multidisciplinarity and patient-centered pathways from diagnosis to treatment, to meet aspiration for comprehensive cancer control. The specialists recommended that the breast radiology team should perform clinical examination, mammography, ultrasound and Doppler ultrasound of the breast and axilla, breast magnetic resonance imaging (MRI) and biopsy under mammography, ultrasound, or MRI guidance after hospitalization (1). Accurately differentiating breast cancer from benign lesions is also important and challenging for clinicians using ultrasound or conventional mammography, especially in dense fibroglandular breasts (2). Breast MRI has been increasingly used in the detection and diagnosis of breast lesions in high-risk patients. Dynamic contrast-enhanced magnetic resonance imaging (DCE-MRI) has become the routine MRI protocol; this technique describes the breast lesions based on their morphological and hemodynamic features. A meta-analysis by Bennani-Baiti et al. (3) included studies applying DCE-MRI as an adjunct to conventional imaging (mammography or ultrasound) to clarify equivocal findings without microcalcifications. The results demonstrate breast MRI as an excellent diagnostic performance with a pooled sensitivity of 99% and specificity of 89%. Another meta-analysis further suggested that breast MRI should be considered for BI-RADS 4 rather than 3 and 5 mammographic microcalcifications, and the presence or absence of enhancement helps to rule out malignancy in mammographic microcalcifications at breast MRI (4). However, the specificity is variable due to background parenchymal enhancement and overlap of kinetic enhancement patterns between benign and malignant breast lesions. The false-positive findings may cause additional examinations or unnecessary surgery (5).

Diffusion-weighted imaging (DWI) has become a promising technique in the differential diagnosis of breast lesions, which allows the measurement of water molecular movement using apparent diffusion coefficient (ADC) values. The monoexponential model in conventional DWI assumed that the microenvironment is homogeneous and that the diffusion of water molecules follows a Gaussian distribution, which causes a linear decay of the logarithm of the DWI signal intensity as the *b*-value increases. The international EUSOBI working group has confirmed the importance of breast DWI in the multiparametric breast MRI protocol to differentiate between benign and malignant breast lesions, distinguish *in situ* from invasive lesions, and predict the responses to and monitor the effects of neoadjuvant therapy over time. The group recommended a high *b*-value of 800 s/mm^2^ and utilization of three orthogonal directions are optimal options to acquire breast DWI. Besides, the ADC should be calculated with a small region of interest on the darkest part of the lesion on the ADC map, avoiding necrotic, noisy, or non-enhancing lesion voxels (6). In the study of Kishimoto et al. (7), they explored the performance of high-resolution DWI in visualizing breast cancer and their extent using readout-segmented echo-planar imaging, and found that malignant mass lesions were depicted with excellent agreement with the pathological results, but half of the non-mass lesions cannot be identified. Besides, due to the natural barriers from cell membranes and cellular compartments that restrict water movement, the logarithmic signal intensity decay will deviate from the plot of the monoexponential model, especially in a high *b*-value range (8).

Beyond the conventional DWI, advanced DWI models such as intravoxel incoherent motion (IVIM) DWI, non-Gaussian DWI, and diffusion tensor imaging (DTI) are increasingly used in this area, allowing the characterization of tissue perfusion and architecture and improving diagnostic performance without the administration of contrast agent (9). Besides the *b*-value, the diffusion MR parameters were also found to be closely correlated with diffusion time in a preclinical study, which should be taken into consideration when interpreting DWI data (10). Diffusion kurtosis imaging (DKI) is an extension of conventional DWI. Jensen et al. (11) first introduced the DKI model in 2005 and developed new fields in brain, liver, and prostate imaging afterward (12–14). The investigation of breast tumors using the DKI model reached a peak in 2019 and became an important research area thereafter (8, 15–20). The model provides microscopic information regarding the deviation of water diffusion from Gaussian distribution with the mean kurtosis (MK) and mean diffusivity (MD), a kurtosis-corrected diffusion coefficient. Interestingly, a study investigated the variability of DKI and IVIM-DWI measurements with different numbers of *b*-values and excitations in the breast found the numbers of *b*-values and excitations performed insignificant impacts on the DKI metrics (21). Their further research indicated that a combination of those two diffusion imaging may provide BI-RADS-equivalent scores almost comparable to BI-RADS category, revealing a bright prospect for DKI studies (22). Previous studies have suggested that DKI has higher specificity for differentiation of malignant and benign breast lesions than conventional DWI (23, 24). However, the diagnostic performance of DKI in the breast was not consistent, and its applications remain debatable. For example, most studies (8, 18, 20) have suggested that breast cancer has a higher MK and a lower MD than benign lesions, while Park et al. (16) reported that the difference in the MK between them is insignificant. Some studies (25–27) have reported that the DKI-derived MK or MD manifested better diagnostic performance than the DWI-derived ADC, while Palm et al. (15) found that DKI did not improve the differentiation performance for breast lesions in clinical protocols. Finally, the sample sizes in most studies were still too small to draw a robust conclusion about the performance of DKI. Therefore, we attempted to pool all the published results about the diagnostic performance of DKI in the differentiation of malignant and benign breast lesions using a meta-analysis method. The controversial issues between different studies will be addressed with more reliable evidence.

## Materials and Methods

### Data Sources

This meta-analysis adhered to the Preferred Reporting Items for Systematic Reviews and Meta-Analyses (PRISMA), and each item listed in the guideline has been checked in our meta-analysis. Studies regarding the differential diagnosis of breast tumors using DKI-derived parameters were systemically retrieved by two senior librarians from the PubMed, Embase, and Web of Science databases without a time limit. A search formula was developed with different combinations of the medical subject headings or keywords for DKI, diffusion kurtosis imaging, non-Gaussian diffusion, and breast or breast lesion/cancer/carcinoma. The primary searches were limited to the titles and abstracts. We also performed a manual retrieval of the reference lists from the included studies.

### Study Selection

Studies that met the following criteria were included: (a) the research purpose was to differentiate malignant and benign breast lesions using DKI parameters; (b) the mean and standard deviation (SD) of each parameter were provided; (c) their diagnostic performance in terms of sensitivity and specificity or true-positive (TP), false-negative (FN), false-positive (FP), and true-negative (TN) rates were reported; and (d) breast cancer was confirmed by pathology after the initial MRI examination. The exclusion criteria mainly were as follows: (a) duplication from the same authors or institutions; (b) meta-analyses, conference abstracts, reviews, or any unpublished results; (c) animal experiments or non-breast research; and (d) non-English studies.

### Data Extraction

One author used a spreadsheet to extract the mean and SD as well as the diagnostic performance of the MK, MD, and ADC with the threshold, area under the curve (AUC), sensitivity, specificity, or TP, FN, FP, and TN values from each study, and this spreadsheet was reviewed by another author. Other information including the first author, year of publication, countries, field strength and vendors, *b*-values in DKI and DWI, patient ages, tumor sizes, and published journal was also collected. The TP, FN, FP, and TN rates were calculated when only the number of malignant and benign lesions and the sensitivity and specificity or receiver operating curve (ROC) was provided.

### Quality Assessment

The quality of studies and likelihood of bias were evaluated using Review Manager 5.3 software (Cochrane Collaboration, Oxford, UK), referring to the Quality Assessment of Diagnostic Accuracy Studies-2 (28). We assessed the risk of bias and applicability in four domains, including patient selection, index tests, reference standard, flow, and timing (29).

### Publication Bias and Heterogeneity Evaluation

As two datasets were pooled in our study, including the quantitative values and diagnostic performance of each parameter, funnel plots, and Begg's test were used to visually and quantitatively assess the publication bias for continuous variables, and Deeks' plot was used to assess the publication bias of sensitivity and specificity using Stata version 12.0 (StataCorp LP, College Station, TX) (30). An asymmetric or skewed funnel plot with *P* < 0.05 for Begg's test or Deeks' test indicated the potential of publication bias (26). The inconsistency index (*I*^2^) and Cochran's *Q*-tests were used to explore the heterogeneity of the included studies, with *I*^2^ > 50% or *P* < 0.05 for Cochran's *Q*-test suggesting statistically significant heterogeneity, in which case a random-effects model was applied in subsequent pooling; a fixed-effects model was applied when *I*^2^ < 50% (31).

### Data Synthesis

We constructed the forest plots for continuous variables and calculated the standardized mean difference (SMD) between malignant and benign breast lesions using Review Manager software. We developed a bivariate regression model to pool the diagnostic performance with the sensitivity, specificity, positive likelihood ratio (PLR), negative likelihood ratio (NLR), diagnostic odds ratio (DOR), and AUC using Stata version 12.0. The summary receiver operating characteristic curves and Fagan's nomograms were also plotted to determine the diagnostic values and predict the post-test probabilities of the MK, MD, and ADC in the differential diagnosis of breast tumors.

## Results

### Literature Search and Selection

**Figure 1:**
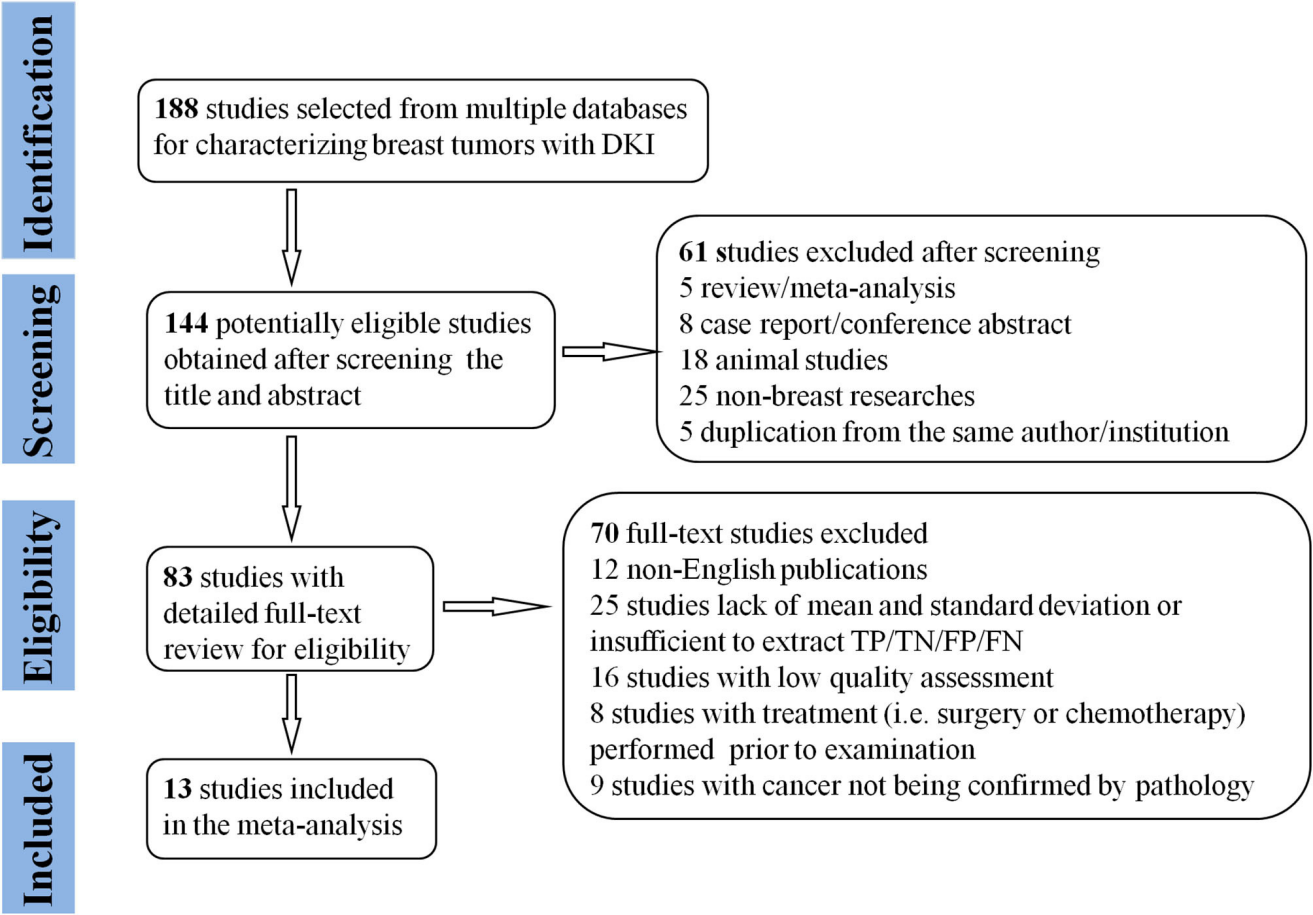
Flowchart detailing the study selection process. Thirteen studies that met the inclusion criteria were included. FN, false negative; FP, false positive; TN, true negative; TP, true positive.

By searching for keywords in the titles and abstracts, a total of 188 potential studies were obtained from multiple databases. Thirteen studies including meta-analyses, conference abstracts, and reviews were excluded after screening the titles and abstracts. Animal studies, non-breast studies, and duplicated studies from the same authors or institutions led to further exclusion of 48 studies. We scrutinized the full texts of the remaining 83 studies in detail and excluded an additional 70 studies for the following reasons: (a) non-English studies; (b) lack of sufficient data to be pooled; (c) low quality assessment; (d) interference of treatment with DKI; and (e) cancer not confirmed by pathology. Eventually, 13 eligible studies with 867 malignant and 460 benign breast lesions were included for analysis. The flowchart detailing the process of study selection is provided in Figure 1. The basic information and diagnostic performance for each included study is detailed in Tables 1, 2. The types of breast cancer mainly included ductal carcinoma *in situ* (DCIS), lobular carcinoma *in situ*, invasive papillary carcinoma, invasive ductal carcinoma (IDC), and mucinous cancer. Benign lesions consisted of fibroadenomas, intraductal papillomas, granulomatous mastitis, epithelial proliferative lesions, fibrocystic changes, phyllodes tumors, and benign breast tissue.

**Table 1:** Basic information for each included study.

**Study**	**Year**	**Country**	**Machine type**	**DKI *b*-values (s/mm^**2**^)**	**DWI *b*-values (s/mm^**2**^)**	**Age (years)**	**Tumor diameters(mm)**	**Malignant**	**Benign**	**Journal**
Wu et al. (32)	2014	China	3T Siemens	0, 250, 500, 750, 1000, 1,500, 2,000	NA	57 ± 14	Benign: 11.4 ± 3.4; Malignant: 35.8 ± 20.1	82	42	*PLoS One*
Nogueira et al. (24)	2014	Portugal	3T Siemens	50, 200, 400, 600, 800, 1,000, 2,000, 3,000	50, 1,000	NA	NA	31	13	*Eur Radiol*
Sun et al. (23)	2015	China	1.5T Siemens	0, 700, 1,400, 2,100, 2,800	50, 1,000	Benign: 36.9 ± 12.2; Malignant: 51.6 ± 10.1	Benign: 19 ± 10; Malignant: 24 ± 10	57	41	*Radiology*
Christou et al. (25)	2017	UK	1.5T GE	0, 400, 800, 1,100, 1,300	NA	54 (37–71)	Benign: 20.8 (10.1–31.5); Malignant: 26.4 (10.5–42.3)	34	19	*Br J Radiol*
Suo et al. (27)	2017	China	3T Philips	0, 10, 30, 50, 100, 150, 200, 500, 800, 1,000, 1,500, 2,000, 2,500	0, 1,000	46 (20–79)	NA	57	44	*J Magn Reson Imaging*
Iima et al. (22)	2018	Japan	3T Siemens	5, 50, 70, 100, 200, 400, 600, 800, 1,000, 1,500, 2,000, 2,500	0, 800	58.5 (20–88)	Benign: 25.7 (10–100); Malignant: 18.2 (10–62)	152	47	*Radiology*
Park et al. (16)	2019	Korea	3T Philips	50, 600, 1,000, 3,000	50, 600, 1,000	46 (29–65)	Benign: 12 (10–50); Malignant: 13.5 (10–92)	30	23	*Magn Reson* *Imaging*
Liu et al. (8)	2019	China	3T Philips	0, 500, 800, 2,000	0, 800	41 (13–64)	NA	42	30	*Eur J Radiol*
Palm et al. (15)	2019	Germany	3T Siemens	50, 750, 1,500	50, 750	NA	NA	68	73	*Magn Reson* *Imaging*
Borlinhas et al. (19)	2019	Portugal	3T Philips	0, 50, 200, 750, 1,000, 2,000	0, 1,000	62 (32–88)	NA	114	0	*Australas Phys* *Eng* *Sci Med*
Huang et al. (20)	2019	China	3T GE	0, 500, 1,000, 1,500, 2,000, 2,500	0, 800	Benign: 34 ± 6; Malignant: 47 ± 12	Benign: 17.9 ± 7.3; Malignant: 26.1 ± 12.3	50	26	*J Magn Reson Imaging*
Li et al. (18)	2020	China	3T Philips	0, 500, 1,000, 1,500, 2,000, 2,500, 3,000	0, 1,000	Benign: 38.9 ± 9.7; Malignant: 55.0 ± 11.5	NA	62	58	*J Magn Reson* *Imaging*
Zhou et al. (33)	2020	China	1.5T Siemens	0, 600, 1,200, 1,800, 2,400	50, 1,000	46.7 ± 15.9	NA	88	44	*J Xray Sci* *Technol*

*NA, not available; DKI, diffusion kurtosis imaging; DWI, diffusion-weighted imaging*.

**Table 2:** The diagnostic performance for each included study.

**Indicators**	**Study**	**Year**	**Sensitivity**	**Specificity**	**AUC**	**TP**	**FP**	**FN**	**TN**	**Threshold**
MK	Wu et al. (32)	2014	0.842	0.929	0.92	69	3	13	39	0.69
	Sun et al. (23)	2015	0.95	0.93	0.974	54	3	3	38	0.8
	Suo et al. (27)	2017	0.86	0.796	0.878	49	9	8	35	0.78
	Christou et al. (25)	2017	0.971	0.937	0.976	33	1	1	18	0.71
	Huang et al. (20)	2018	0.94	0.9231	0.979	47	2	3	24	1.05
	Palm et al. (15)	2019	0.93	0.82	0.89	63	13	5	60	0.69
	Liu et al. (8)	2019	0.8333	0.8333	0.875	35	5	7	25	NA
	Li et al. (18)	2020	0.71	0.862	0.821	44	8	18	50	0.712
	Zhou et al. (33)	2020	0.915	0.853	0.911	81	6	7	38	0.775
MD	Wu et al. (32)	2014	0.793	0.929	0.86	65	3	17	39	1.58
	Sun et al. (23)	2015	0.97	0.88	0.973	55	5	2	36	1.67
	Suo et al. (27)	2017	0.825	0.818	0.876	47	8	10	36	1.29
	Christou et al. (25)	2017	0.912	0.937	0.949	31	1	3	18	1.57
	Huang et al. (20)	2018	0.94	0.808	0.928	47	5	3	21	1.406
	Palm et al. (15)	2019	0.94	0.82	0.91	64	13	4	60	1.68
	Park et al. (16)	2019	0.625	0.913	0.755	19	2	11	21	1.065
	Liu et al. (8)	2019	0.8333	0.7	0.749	35	9	7	21	NA
	Li et al. (18)	2020	0.597	0.897	0.729	37	6	25	52	1.335
	Zhou et al. (33)	2020	0.822	0.983	0.936	72	1	16	43	1.475
ADC	Sun et al. (23)	2015	0.86	0.83	0.895	49	7	8	34	1.211
	Suo et al. (27)	2017	0.93	0.75	0.897	53	11	4	33	0.87
	Huang et al. (20)	2018	0.96	0.7692	0.911	48	6	2	20	1.15
	Palm et al. (15)	2019	0.96	0.85	0.92	65	11	3	62	1.36
	Park et al. (16)	2019	0.8125	0.7143	0.768	24	7	6	16	1.27
	Liu et al. (8)	2019	0.7381	0.7	0.69	31	9	11	21	NA
	Li et al. (18)	2020	0.629	0.897	0.759	39	6	23	52	1.091
	Zhou et al. (33)	2020	0.783	0.932	0.897	69	3	19	41	1.178

*NA, not available; MK, mean kurtosis; MD, mean diffusivity; ADC, apparent diffusion coefficient; AUC, area under the curve; FN, false negative, FP, false positive; TN, true negative, TP, true positive. Threshold values of ADC and MD are factors of 10^−3^ mm^2^/s. As not all included studies provide the diagnostic performance of MK, MD, and ADC, the number of studies in this table is less than that in Table 1. Besides, if the results from one study significantly deviate from the other studies in the sensitivity analysis/forest plots, that study will also be excluded for analysis due to instability*.

### Quality Assessment

**Figure 2:**
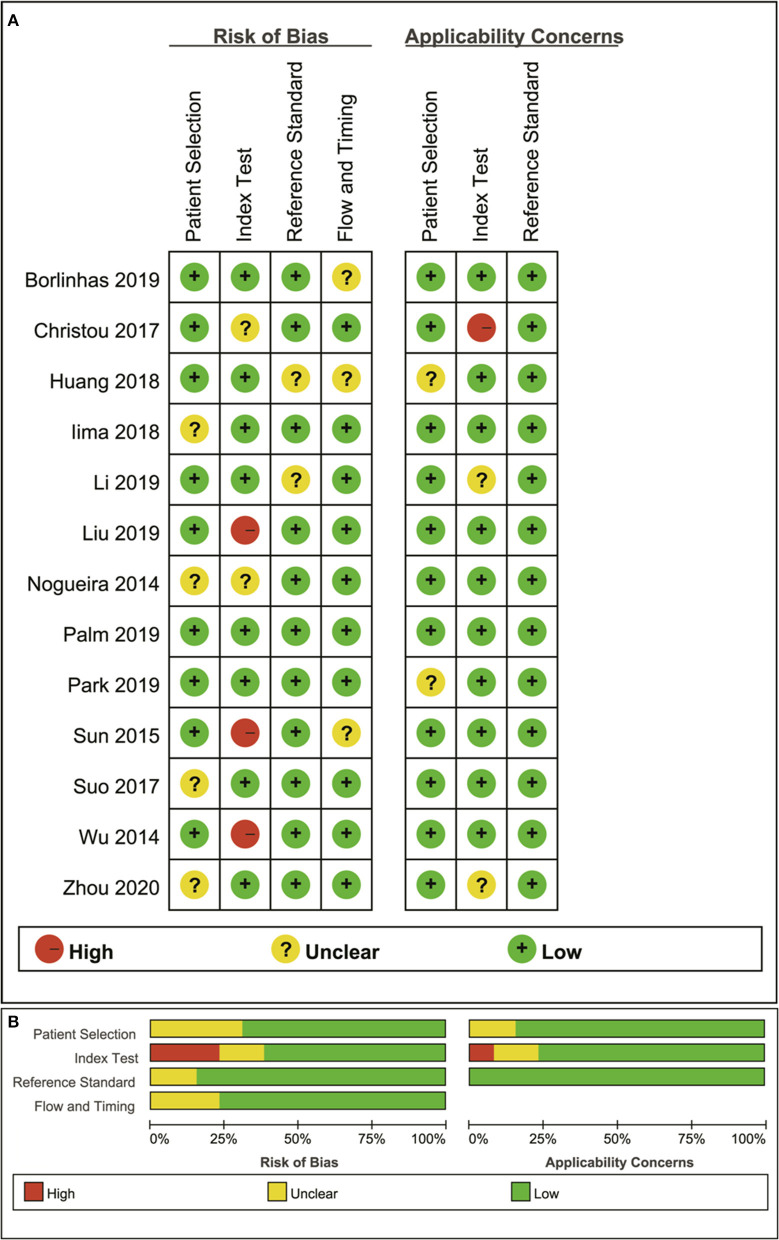
Distribution of the risk of bias and applicability concerns for each included study using QUADAS-2 **(A)** and a summary methodological quality **(B)**.

The distribution of Quality Assessment of Diagnostic Accuracy Studies-2 scores for risk of bias and applicability concerns are shown in Figure 2. The overall quality of included studies was acceptable. Regarding patient selection domain, four studies were considered to have an unclear risk of bias due to a small sample of benign lesions for comparison, and unknown sequence for patient enrollment. The applicability concerns were considered high as the tumor types were inconsistent in the two groups. Five studies were determined to have an unclear or a high risk of bias with high concerns of applicability for the index test as the threshold values for the MK, MD, or ADC were not provided. Two studies showed unclear risks of bias for the reference standard domain because some of the benign lesions were diagnosed through long-term follow-up. Most studies had a low risk of bias in the patient flow and timing domains because of the short time interval between MR examination and pathological confirmation (within 1 week).

### Quantitative Analysis

#### MK Used for the Diagnosis of Breast Tumors

**Figure 3:**
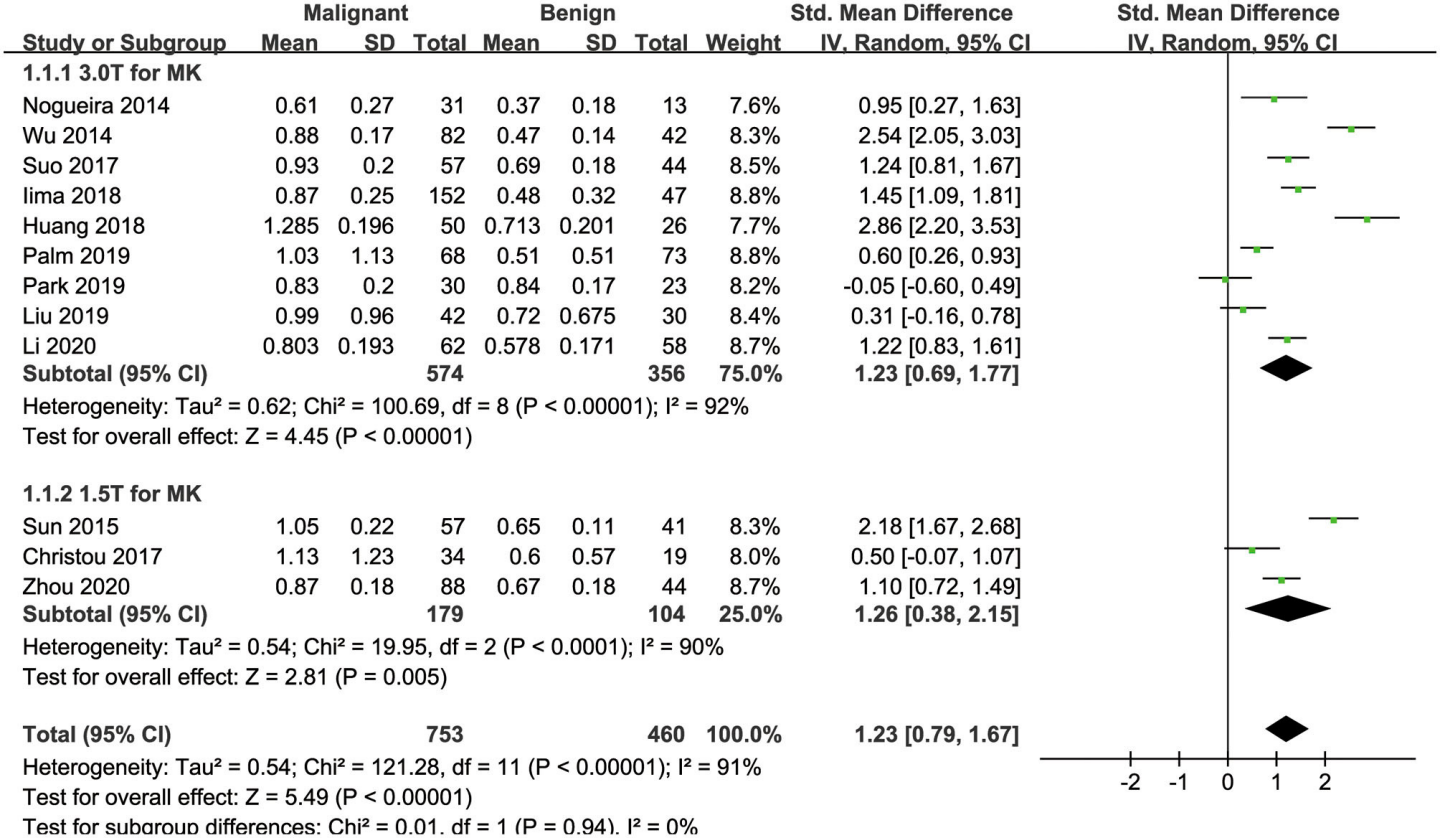
Forest plot of the mean value of the mean kurtosis (MK) between malignant and benign breast lesions. The standardized mean differences indicated that breast cancers had a significantly higher MK than benign lesions.

**Figure 4:**
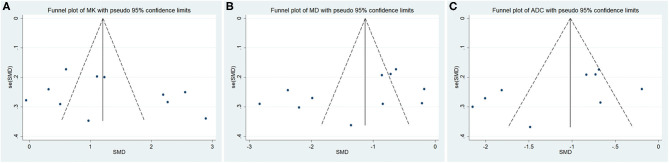
Funnel plot of the **(A)** mean kurtosis (MK), **(B)** mean diffusivity (MD), and **(C)** apparent diffusion coefficient (ADC). No publication bias was observed.

Twelve studies evaluating the MK for diagnosing breast tumors were included for analysis. The results of χ^2^ = 121.28 and *P* < 0.001 for the heterogeneity test with *I*^2^ = 91% suggested high heterogeneity among the included studies. The forest plot in Figure 3 shows the distribution of the MK between malignant and benign breast lesions. A random-effects model generated an SMD of 1.23 (0.79, 1.67) (*P* < 0.001) between malignant and benign breast lesions differentiated by the MK. A basically symmetrical funnel plot, as shown in Figure 4, and *P* = 0.640 of Begg's test suggested no publication bias in the MK.

#### MD Used for the Diagnosis of Breast Tumors

**Figure 5:**
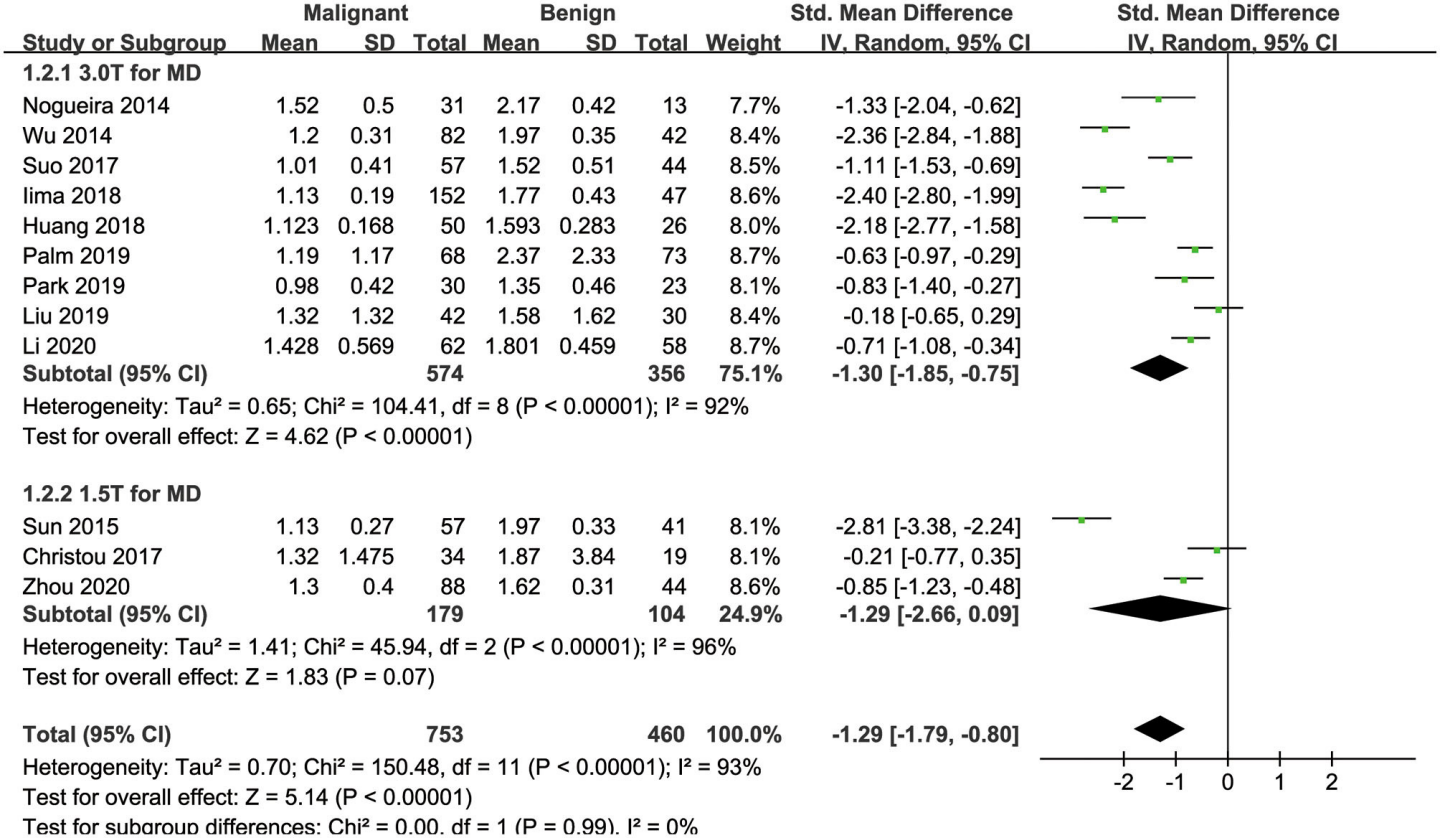
Forest plot of the mean value of the mean diffusivity (MD) between malignant and benign breast lesions. The standardized mean differences indicated that breast cancers had a significantly lower MD than benign lesions.

Twelve studies regarding the MD applied in diagnosing breast tumors were included for analysis. The results of χ^2^ = 150.48 and *P* < 0.001 for the heterogeneity test with *I*^2^ = 93% suggested high heterogeneity among the included studies. The forest plot in Figure 5 shows the distribution of the MD between malignant and benign breast lesions. A random-effects model generated an SMD of −1.29 (−1.79, −0.80) (*P* < 0.001) between malignant and benign breast lesions differentiated by the MD. A symmetrical funnel plot, as shown in Figure 4, and *P* = 0.161 of Begg's test suggested no publication bias in the MD.

#### ADC Used for the Diagnosis of Breast Tumors

**Figure 6:**
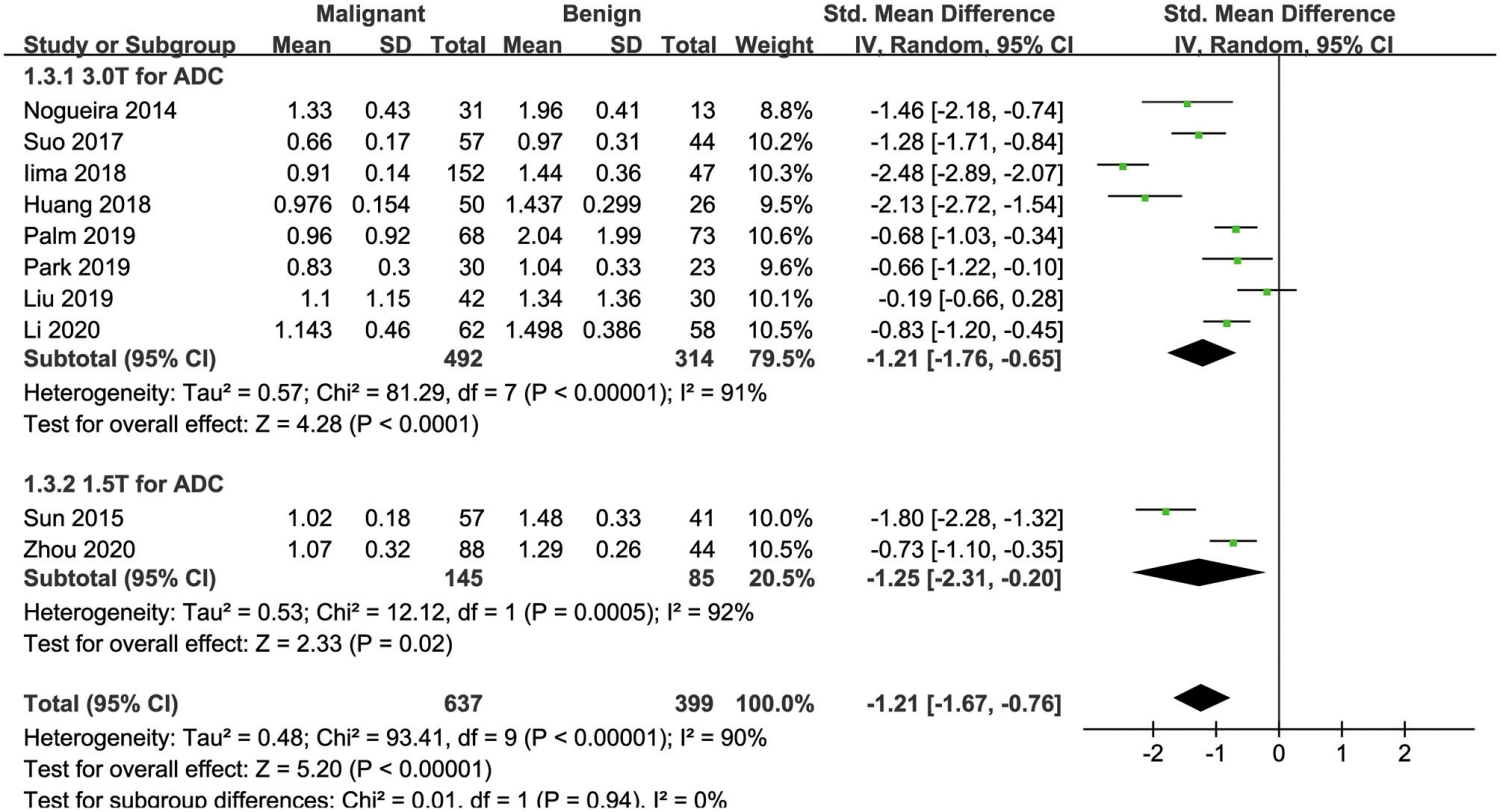
Forest plot of the mean value of the apparent diffusion coefficient (ADC) between malignant and benign breast lesions. The standardized mean differences indicated that breast cancers had a significantly lower ADC than benign lesions.

Ten of the included studies regarding the ADC applied for diagnosing breast tumors were pooled. The results of χ^2^ = 93.41 and *P* < 0.001 for the heterogeneity test with *I*^2^ = 90% suggested high heterogeneity among the pooled studies. The forest plot in Figure 6 shows the distribution of the ADC between malignant and benign breast lesions. A random-effects model generated an SMD of −1.21 (−1.67, −0.76) (*P* < 0.001) between malignant and benign breast lesions differentiated by the ADC. A symmetric funnel plot, as shown in Figure 4, and *P* = 0.076 of Begg's test suggested no publication bias in the ADC.

### Subgroup Analysis

Table 3 shows the subgroup analyses to explore the influence of ethnicity, vender, field strength, number of *b*-value, and study design in the pooled results. The results of heterogeneity analysis for each subgroup are also listed in the table. Figures 3, 5, 6 included subgroup analyses for field strengths (1.5 T or 3.0 T). A subgroup analysis of MK, MD, and ADC found no statistical difference in GE machine between breast cancer and benign lesions (*P* = 0.16, 0.23, and 0.12 for MK, MD, and ADC). The other subgroups all demonstrated a statistical difference between breast cancer and benign lesions (*P* < 0.05). There is no heterogeneity in the whites for MK and prospective study for MK, MD, and ADC (*I*^2^ < 50%). After comparing the SMD within each subgroup, the ethnicity (*P* = 0.018, 0.023, and 0.012 for MK, MD, and ADC), number of *b*-value (*P* = 0.039, 0.025, and 0.037 for MK, MD, and ADC) and study design (*P* = 0.015, 0.022, and 0.017 for MK, MD, and ADC) introduced a potential heterogeneity in the pooled results. However, different vendors (*P* = 0.18, 0.47, and 0.51 for MK, MD, and ADC) and field strengths (*P* = 0.94, 0.99, and 0.94 for MK, MD, and ADC) manifested a stable SMD in the subgroup analyses.

**Table 3:** Subgroup analyses for the MK, MD, and ADC values in the differential diagnosis of breast lesions.

	**MK**				**MD**				**ADC**			
	**Studies**	**SMD**	***P***	***I*^**2**^**	**Studies**	**SMD**	***P***	***I*^**2**^**	**Studies**	**SMD**	***P***	***I*^**2**^**
**Ethnicity**												
Whites	3	0.63 (0.36, 0.90)	0.001	0	3	−0.68 (−1.20, −0.16)	0.01	66%	2	−0.73 (−1.75, −0.26)	0.008	72%
Asians	9	1.42 (0.88, 1.95)	0.001	92%	9	−1.48 (−2.08, −0.89)	0.001	93%	8	−1.26 (−1.81, −0.71)	0.001	92%
**Vendor**												
Siemens	6	1.46 (0.88, 2.05)	0.001	91%	6	−1.72 (−2.50, −0.95)	0.001	94%	5	−1.42 (−2.16, −0.68)	0.001	93%
GE	2	1.68 (−0.64, 3.99)	0.16	96%	2	−1.19 (−3.12, 0.73)	0.23	95%	2	−1.93 (−2.30, −1.56)	0.12	56%
Philips	4	1.70 (0.08, 1.32)	0.03	86%	4	−1.71 (−1.09, −0.33)	0.001	65%	4	−1.75 (−1.19, −0.31)	0.001	74%
**Field strength**												
1.5 T	3	1.26 (0.38, 2.15)	0.001	90%	3	−1.29 (−2.66, −0.09)	0.001	96%	3	−1.25 (−2.31, −0.20)	0.001	92%
3.0 T	10	1.23 (0.69, 1.77)	0.001	92%	10	−1.30 (−1.85, −0.75)	0.001	92%	10	−1.21 (−1.76, −0.65)	0.001	91%
**No. of** ***b*****-value**												
*n* ≤ 5	6	0.78 (0.21, 1.34)	0.007	89%	6	−0.91 (−1.56, −0.26)	0.006	92%	6	−0.81 (−1.29, −0.33)	0.001	83%
*n* > 5	6	1.70 (1.16, 2.23)	0.001	87%	6	−1.68 (−2.32, −1.03)	0.001	81%	5	−1.63 (−2.30, −0.96)	0.001	90%
**Study design**												
Prospective	5	1.81 (0.94, 2.68)	0.001	21%	5	−1.78 (−2.70, −0.87)	0.001	13%	3	−1.83 (−2.16, −1.50)	0.001	2%
Retrospective	7	0.86 (0.48, 1.24)	0.001	83%	7	−0.96 (−1.47, −0.45)	0.001	91%	7	−0.98 (−1.52, −0.45)	0.001	92%

*SMD, standardized mean difference; MK, mean kurtosis; MD, mean diffusivity; ADC, apparent diffusion coefficient; I^2^, inconsistency index*.

### Differentiation Between DCIS and IDC

The MK from three studies (19, 24, 27), MD from four studies (15, 16, 24, 27), and ADC from four studies (15, 16, 24, 27) used for the differentiation of DCIS and IDC were further pooled. DCIS showed a significantly lower MK than IDC with an SMD of −1.36 (−2.34, −0.38) (*P* = 0.006) and *I*^2^ = 77%. Although DCIS had a higher MD and ADC than IDC, with an SMD of 0.29 (−0.16, 0.73) (*P* = 0.20) and 0.26 (−0.18, 0.71) (*P* = 0.24), respectively, the results were insignificant.

### Diagnostic Performance of the MK, MD, and ADC

**Figure 7:**
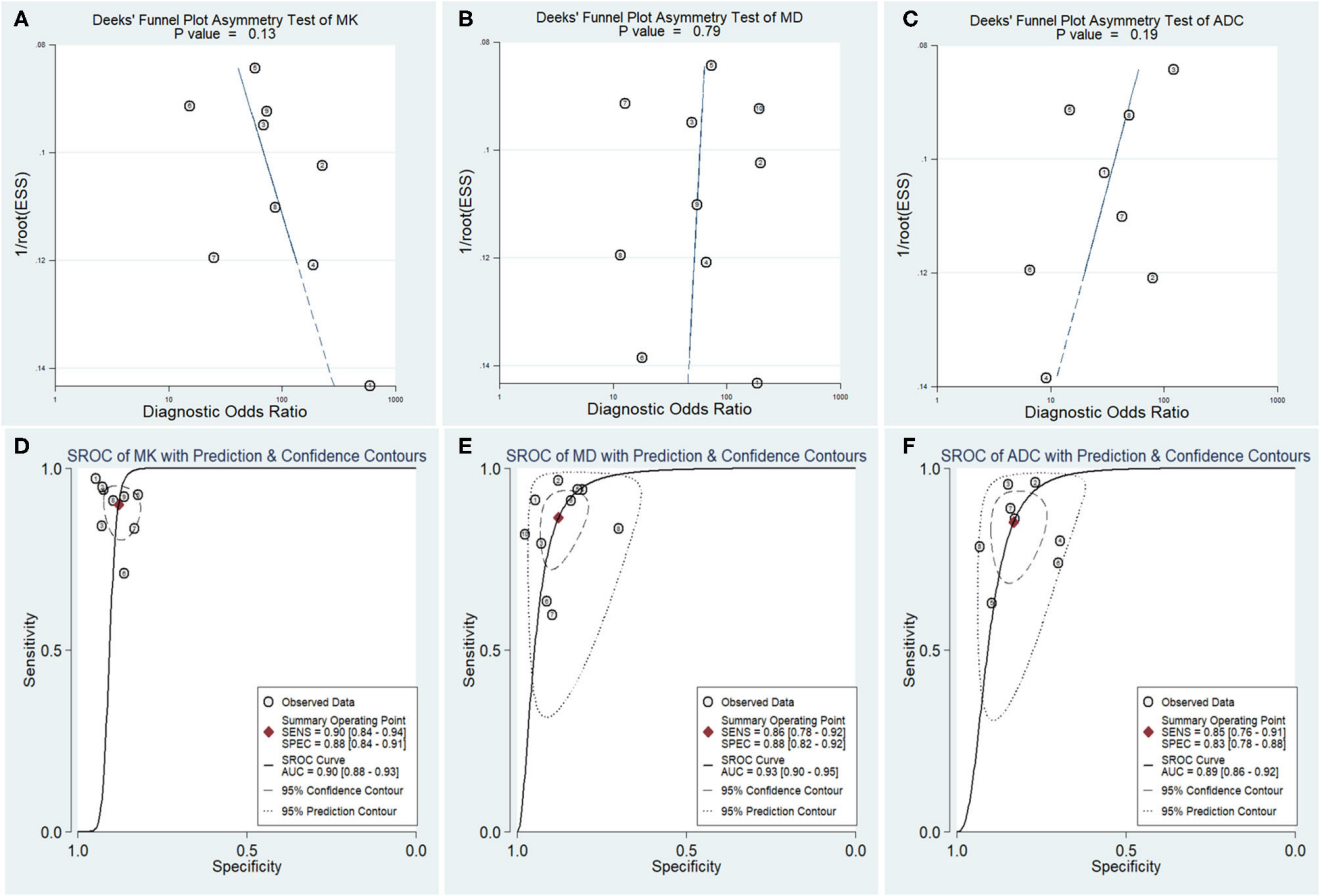
Deeks' funnel plots **(A–C)** and summary receiver operating characteristic **(D–F)** curve of the mean kurtosis (MK), mean diffusivity (MD), and apparent diffusion coefficient (ADC) in the diagnosis of breast lesions. No publication bias was indicated in the three parameters.

The DOR is the ratio of the PLR to the NLR, which reflects the association between the results of a diagnostic test and a suspected disease. The larger the DOR is, the better the differentiation capability of MRI parameters is. The diagnostic performance with the pooled sensitivity, specificity, and DOR of the MK, MD, and ADC is shown in Table 4. Figure 7 shows the Deeks' funnel plots and summary receiver operating characteristic curves of the MK, MD, and ADC. Deeks' funnel plots indicated no obvious publication bias in the MK, MD, and ADC (*P* > 0.05). The DKI-derived MK (sensitivity = 90%, specificity = 88%, DOR = 66) and MD (sensitivity = 86%, specificity = 88%, DOR = 46) showed a comparable sensitivity and specificity with the DWI-derived ADC (sensitivity = 85%, specificity = 83%, DOR = 29) in differentiating malignant from benign breast lesions.

**Table 4:** Pooled estimates and heterogeneity measures for MK, MD, and ADC.

	**Sensitivity**	**Specificity**	**PLR**	**NLR**	**DOR**	**AUC**	***I***^****2****^	
							**Sensitivity**	**Specificity**
MK	0.90 (0.84, 0.94)	0.88 (0.84, 0.91)	7.5 (5.6, 10.1)	0.11 (0.07, 0.18)	66 (35, 125)	0.90 (0.88, 0.93)	74.94%	0
MD	0.86 (0.78, 0.92)	0.88 (0.82, 0.92)	7.1 (5.0, 10.1)	0.16 (0.09, 0.25)	46 (25, 84)	0.93 (0.90, 0.95)	84.25%	52.87%
ADC	0.85 (0.76, 0.91)	0.83 (0.78, 0.88)	5.1 (3.8, 6.9)	0.18 (0.11, 0.29)	29 (15, 54)	0.89 (0.86, 0.92)	80.73%	45.33%

*The data in parentheses indicate mean and 95% confidence intervals. MK, mean kurtosis; MD, mean diffusivity; ADC, apparent diffusion coefficient; PLR, positive likelihood ratio; NLR, negative likelihood ratio; DOR, diagnostic odds ratio; AUC, area under the curve; I^2^, inconsistency index*.

### Post-test Probabilities

**Figure 8:**
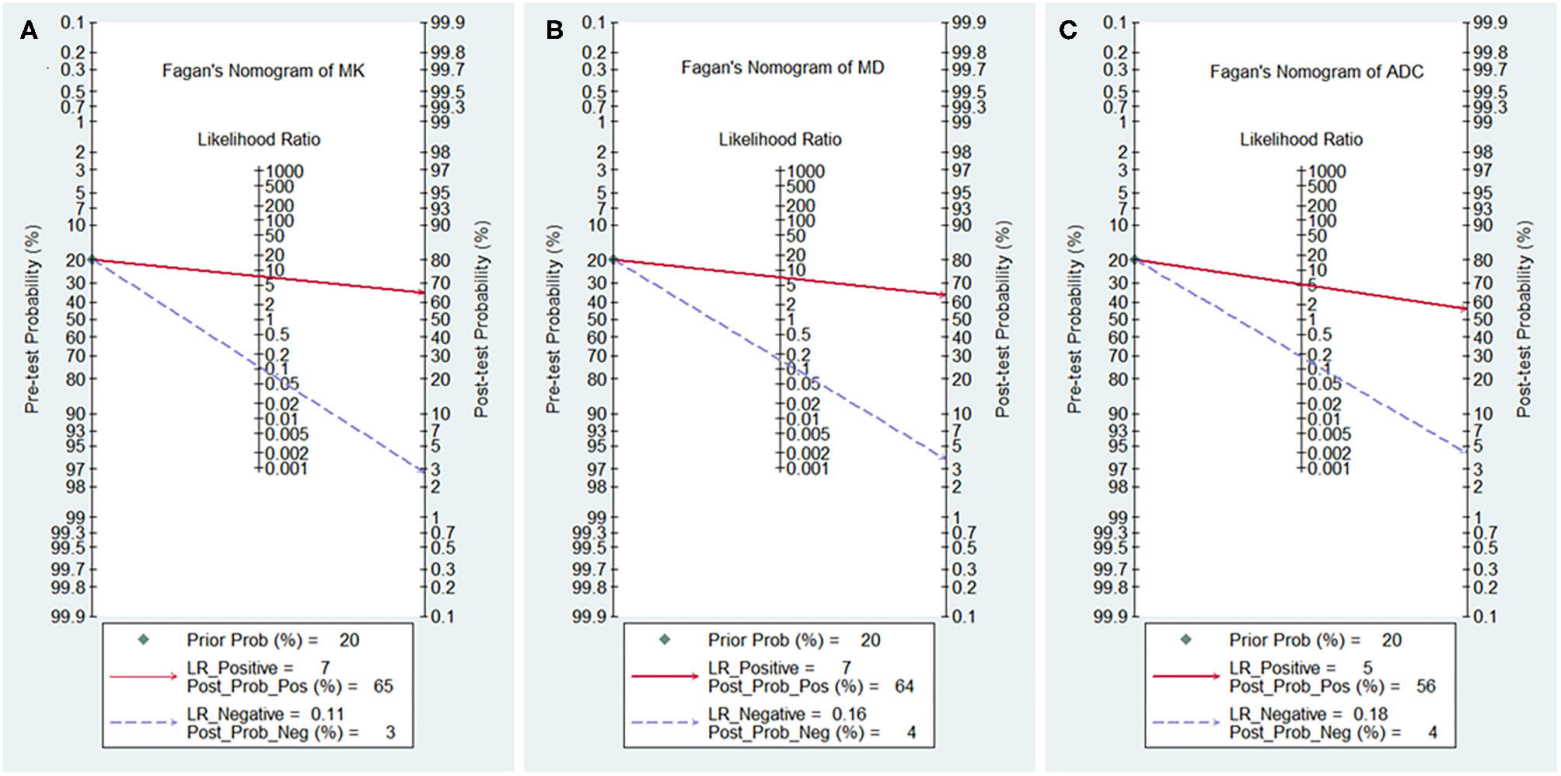
Fagan's nomogram of the **(A)** mean kurtosis (MK), **(B)** mean diffusivity (MD), and **(C)** apparent diffusion coefficient (ADC).

The likelihood ratio and post-test probability are also important for diagnosing a disease (34); these values provide the likelihood that a patient will be diagnosed with a certain disease or not using MRI parameters. Figure 8 shows Fagan's nomograms of the MK, MD, and ADC for predicting post-test probabilities. All the pretest probabilities were set at 20% by default. We regarded the diagnosis of breast cancer as a positive event, corresponding to a higher MK and a lower MD and ADC. Similarly, diagnosing a benign lesion with a lower MK and a higher MD and ADC represented a negative event. The post-test probability increased to 65% from the pretest probability of 20% with a PLR of 7.5 and decreased to 3% with an NLR of 0.11, with the prompt of the MK. This indicated that the diagnostic preference for breast cancer is obviously enhanced with the use of the MK (a higher MK) compared with the condition without the prompt of the MK, with a diagnostic probability set at 20% beforehand. In contrast, the probability of diagnosing breast cancer significantly decreases from 20 to 3% when a negative event occurs (a lower MK). Similarly, the post-test probability of diagnosing breast cancer reaches 64% with a PLR of 7.1 and decreases to 4% with an NLR of 0.16 using the MD for guidance. The post-test probability of diagnosing breast cancer reaches 56% with a PLR of 5.1 and decreases to 4% with an NLR of 0.18 with the use of the ADC. These data indicate that DKI parameters helped to enhance the accuracy for diagnosing breast cancer.

## Discussion

DKI is a non-Gaussian diffusion-weighted analysis method and includes calculation of diffusivity in various tissues. It has been regarded as a complementary approach to improve the diagnostic performance of breast DCE-MRI, especially for increasing its specificity (16). A previous meta-analysis confirmed the value of DKI in grading glioma with a good pooled sensitivity of 0.85 and a specificity of 0.92 (35). To our knowledge, there is still no study with a large sample size to determine the value of DKI for quantitatively distinguishing breast cancer from benign lesions in the background of DKI becoming a research focus in whole-body tumors. Our study provided a timely summary of this issue through pooling all the published evidence with strict inclusion criteria and quality assessments. The results showed a promising prospect for DKI to be incorporated into the MRI protocol for evaluating the breast.

In our study, the SMDs suggested that breast cancer demonstrated a higher MK and a lower MD and ADC than benign lesions. Breast cancer usually demonstrates dense cellularity with an active proliferation capacity; the extracellular space is often infiltrated by inflammatory cells, which may reduce the extracellular space and limit the diffusion of water molecules, causing a reduction in the diffusion coefficient. The pooled results also suggested that the DKI-derived MK and MD provided improved diagnostic performance with a higher specificity, DOR, and post-test probability; however, they were not overwhelmingly superior to DWI-derived ADC based on the AUCs. In a previous study, Iima et al. (36) used non-Gaussian diffusion and IVIM-DWI to diagnose breast lesions and found that the AUC of ADC0 was significantly higher than that of MK, suggesting the superiority of conventional DWI and it having more clinical availability. A complex and heterogeneous microstructure is a common feature of malignant tumors arising from necrosis, cancer nests, intraductal components, and plenty of barriers and compartmentalization between or within the cells (18). The diffusion of water molecules in this microenvironment will deviate from the mono-exponential Gaussian model at high *b*-values, leading to inaccurate fitting and calculation of the diffusion coefficient. Considering the above factors, a DKI non-Gaussian model was developed and showed improved diagnostic performance for prostate cancer (37), hepatocellular carcinoma (38), and glioma (39) as well as for breast tumors in our study. DKI can add valuable implications of microstructural changes to the findings of conventional DCE-MRI, whose pooled specificity was only 71% in a previous meta-analysis (40).

As the treatment strategy is different between DCIS and IDC, we further pooled the three parameters for the differentiation between DCIS and IDC. The results suggested that IDC had a higher MK and a lower MD and ADC than DCIS, with only a significant difference in the MK. Given the small number of studies, the current results were not robust. Some studies have also indicated that these three parameters can further identify the histological grades, Ki-67 expression, and lymph node status instead of hormone receptor status among different subtypes of breast cancer (20, 23). More studies should be included in the future.

The MK, MD, and ADC all demonstrated obvious heterogeneity, which should be explored. First, the combination of *b*-values including the number and the highest *b*-value varied considerably among studies. The subgroup analysis of MK, MD, and ADC between the two groups also confirmed that the number of *b*-value may have varied the results significantly. This indicated that a uniform combination of *b*-values with relatively high specificity but less scanning times should be standardized for clinical use. Second, both 1.5-T and 3.0-T MR scanners were used to perform DKI in these studies. Theoretically, a lower field strength may decrease the signal intensity at high *b*-values, which are essential for DKI. However, different vendors and field strengths manifested a stable SMD in the subgroup analyses. This indicated that the DKI data generated from different vendors and field strengths are comparable and reliable. Third, subgroup analysis also demonstrated that the study design was a source of heterogeneity in the pooled SMDs. A prospective study that may be performed in more consistent conditions indicated a lower heterogeneity in MK, MD, and ADC. Fourth, the SMDs of MK, MD, and ADC between the Whites and Asians were significantly different, indicating that the comparison between different ethnicities should be cautious. Although we have performed subgroup analyses to explore the heterogeneity, the heterogeneity is still high in some groups. The post-processing methods were different; some studies performed histogram analyses for all the lesions, while others delineated the lesions at the largest section as the region of interest. The tumor types were inconsistent in the two groups and may have had different biological characteristics that altered the DKI values.

There was one important limitation. Most of the included studies only used three orthogonal directions to sensitize diffusion gradients in DKI, which can only evaluate the tissue complexity without a direction feature. DTI applies at least six gradient directions to generate eigenvectors that describe water diffusion in a certain direction and help to recognize ductal or glandular tissues. A previous meta-analysis confirmed that DTI with fractional anisotropy also demonstrated superior diagnostic performance in the differential diagnosis of breast lesions (34). A combination of these techniques may further improve the specificity in characterizing breast lesions.

## Conclusions

The DKI-derived MK and MD demonstrate a comparable diagnostic performance in the discrimination of breast tumors based on their microstructural and non-Gaussian characteristics but are not superior to ADC. The MK rather than the MD and ADC can further differentiate IDC from DCIS. MK, MD, and ADC demonstrate potential to identify the histological grades, Ki-67 expression, and lymph node status among different subtypes of breast cancer, but more studies should be included in the future.

## Data Availability Statement

All datasets presented in this study are included in the article/supplementary material.

## Author Contributions

JL, CX, and XL conceived the study and revised the manuscript. ZL and XML drafted the manuscript. WD and CP searched the databases and acquired the data. JL and HH performed data analysis and interpretation. All authors contributed substantially to the preparation of the manuscript.

## Conflict of Interest

The authors declare that the research was conducted in the absence of any commercial or financial relationships that could be construed as a potential conflict of interest.

## Abbreviations

AUC: area under the curve
ADC: apparent diffusion coefficient
DKI: diffusion kurtosis imaging
DOR: diagnostic odds ratio
DCIS: ductal carcinoma *in situ*
IDC: invasive ductal carcinoma
MK: mean kurtosis
MD: mean diffusivity
MRI: magnetic resonance imaging
NLR: negative likelihood ratio
SMD: standardized mean difference
*I*^2^: inconsistency index
PLR: positive likelihood ratio.
